# SATISFACTION, EFFECTIVENESS, AND USABILITY OF TELEREHABILITATION FOR PARKINSON’S DISEASE PATIENTS

**DOI:** 10.2340/jrm.v57.39819

**Published:** 2025-01-03

**Authors:** Shohei OKUSA, Hiroki SAEGUSA, Kazuya MIYAKAWA, Yuta TSUTSUMI, Sae ISHIDA, Kyoko NISHIKATA, Tomonori NUKARIYA, Toshiki TEZUKA, Yoshihiro NIHEI, Yasuhiro KITAGAWA, Shin-ichiro KUBO, Norihiro SUZUKI, Jin NAKAHARA, Morinobu SEKI

**Affiliations:** 1 Department of Neurology, Keio University School of Medicine, Tokyo; 2 Department of Neurology, Shonan Keiiku Hospital, Kanagawa; 3 Department of Rehabilitation, Shonan Keiiku Hospital, Kanagawa; 4 Department of Orthopedics, Shonan Keiiku Hospital, Kanagawa; 5 Department of Neurology, Eisei Hospital, Tokyo; 6 Parkinson’s Disease Center, Keio University Hospital, Tokyo, Japan

**Keywords:** Parkinson’s disease, telerehabilitation, telehealth usability questionnaire, coronavirus disease 2019

## Abstract

**Objective:**

To evaluate the satisfaction, effectiveness, and usability of a telerehabilitation programme for Parkinson’s disease (PD) patients.

**Design:**

Prospective cohort study.

**Subjects/Patients:**

PD patients based on the diagnostic criteria for clinically established or probable PD published by the International Parkinson and Movement Disorder Society.

**Methods:**

The telerehabilitation was conducted twice a week via a Zoom meeting platform, using pre-recorded rehabilitation contents shared during the sessions. In this study we administered several questionnaires, i.e., a self-report questionnaire on the effectiveness of telerehabilitation, the Parkinson’s Disease Questionnaire-39 (PDQ-39), and the Telehealth Usability Questionnaire (TUQ), in order to evaluate the satisfaction, effectiveness, and usability of our telerehabilitation programme.

**Results:**

Fifty-six PD patients were included in the analysis. After 6 months, 91.1% expressed satisfaction with the telerehabilitation and 91.9% reported telerehabilitation had helped them develop an exercise routine, but the PDQ-39 showed no significant improvement in quality of life. The TUQ showed higher scores for Usefulness (76.1%), Ease of Use and Learnability (73.5%), Interface Quality (75.4%), and Satisfaction and Future Use (82.2%).

**Conclusion:**

Satisfaction with telerehabilitation was high, particularly with regard to positive effects on emotional well-being. Telerehabilitation usability was also found to be high.

## LAY ABSTRACT

Due to the global outbreak of the coronavirus disease in 2019, many Parkinson’s disease patients were unable to visit rehabilitation centres and expressed a need for rehabilitation that could be done at home. While face-to-face medical care and rehabilitation were constrained, telemedicine through the internet experienced rapid growth and development. Therefore, we launched a telerehabilitation programme that enabled patients to engage in rehabilitation at home via the internet using a personal computer or a cellular phone. After 6 months of telerehabilitation, most patients expressed satisfaction with the telerehabilitation and reported telerehabilitation had helped them develop an exercise routine. Also, they recognized the positive impact on their emotional well-being and reported that engaging in telerehabilitation together with other patients suffering from the same illness was a positive experience. They realized high usability, because the access was easy, and no hospital visit was required. Telerehabilitation may be a new treatment option for Parkinson’s disease patients.

Parkinson’s disease (PD) is a movement disorder mainly characterized by bradykinesia, rigidity, tremor, postural instability, and gait disturbance. In recent years, the number of patients diagnosed with PD has been increasing each year, making it extremely important to consider optimal treatment strategies. Treatment should be an appropriate combination of various medications, device-aided therapy, and rehabilitation. Rehabilitation plays an essential role in the treatment of PD patients across all disease stages (1). Rehabilitation has been reported to be effective even for axial symptoms such as dysphagia, hypophonia, postural instability, and freezing of gait, which often exhibit limited responsiveness to pharmacological interventions (2, 3). Moreover, the effectiveness of rehabilitation extends beyond temporary improvement of symptoms, providing long-term benefits (4). Notably, rehabilitation has demonstrated potential in reducing medication dosages (5) and mortality (6).

However, due to the global outbreak of coronavirus disease in 2019 (COVID-19), people have increasingly avoided contact and reduced their opportunities to go out. In a previous study, we investigated the impact of the COVID-19 pandemic on the lives of PD patients (7). The findings revealed that many respondents were unable to visit rehabilitation centres and expressed a need for rehabilitation that could be done at home. While face-to-face medical care and rehabilitation were constrained during the COVID-19 epidemic, telemedicine through the internet experienced rapid growth and development. The effectiveness of remote rehabilitation has been shown to be comparable to that of in-person rehabilitation (8).

In April 2022, we launched a telerehabilitation programme that enabled patients to engage in rehabilitation at home via the internet using a personal computer or a cellular phone. Although some studies have reported the usefulness of telerehabilitation for PD patients (9, 10), there are still few reports on the effectiveness and usability of large-scale and long-term telerehabilitation. Furthermore, there have been no reports evaluating the usefulness of telerehabilitation for PD patients using a specialized questionnaire called the Telehealth Usability Questionnaire (TUQ) (11). The TUQ was developed to examine the usability of telehealth implementation and services. In this study we administered several questionnaires, i.e., a self-report questionnaire on the effectiveness of telerehabilitation, the Parkinson’s Disease Questionnaire-39 (PDQ-39), and the TUQ, in order to evaluate the satisfaction, effectiveness and usability of our telerehabilitation programme.

## METHODS

### Participants

All participants were PD patients attending an outpatient clinic at Keio University Hospital or Shonan Keiiku Hospital, and had been diagnosed with PD based on the diagnostic criteria for clinically established or probable PD published by the International Parkinson and Movement Disorder Society (12). Written consent was obtained from each patient after approval by the Ethics Committee of Shonan Keiiku Hospital (#021-024).

### Telerehabilitation

Rehabilitation sessions were conducted twice a week at 17.00 h on Tuesdays and Thursdays, with each session lasting 40 min. The telerehabilitation programme utilized the Zoom meeting platform (Fig. S1), incorporating pre-recorded rehabilitation contents shared during the sessions. The rehabilitation regimen was primarily centred on chair-based physiotherapy, accompanied by additional training programmes targeting articulation, swallowing, and cognitive functions (Tables SI and SII). The content of the rehabilitation was revised appropriately every 2 weeks. During online sessions, patients participated while keeping their camera on, allowing the physiotherapist to provide real-time voice instructions and guidance on their movements. To ensure safe and effective rehabilitation, we ensured that the patient was in an ON state at the start of telerehabilitation. The initial 20 min of each 40-min session were devoted to rehabilitation exercises, followed by a break during which the physiotherapist provided advice on managing life with PD. At the end of the session, the attending physician gave a concise lecture covering various aspects of PD, encompassing medications, rehabilitation, nutrition, motor and non-motor symptoms, and so on. Technical support on how to operate the telerehabilitation programme was provided in person or by e-mail, but no free hardware or software was provided, nor was any financial assistance given. Also, all telerehabilitation programmes were also offered free of charge.

### Survey

At the beginning of the telerehabilitation, we collected the patient’s background information including age, body mass index (BMI), disease duration, Hoehn and Yahr (HY) stage, presence of comorbidities, and other relevant factors. Additionally, the PDQ-39 was employed to assess quality of life (QOL). Moreover, following a study conducted by Politis et al. (13), patients were asked to select and rank their top 3 most bothersome symptoms from a list of both motor and non-motor symptoms. A total score was then calculated according to their ranking, with the most troublesome symptom assigned 3 points, the second most troublesome symptom 2 points, and the third most troublesome symptom 1 point. After 6 months of the telerehabilitation programme, we administered a self-report questionnaire that we created to evaluate the satisfaction and effectiveness of telerehabilitation. To assess its effectiveness, a list of activities of daily living (ADL), based on 10 items from the Barthel Index, and instrumental activities of daily living (IADL), based on 8 items from the Lawton scale, was prepared. Patients were asked whether they were aware of any improvement, with multiple responses allowed. The PDQ-39 was also re-administered to investigate any changes in the patients’ QOL. Finally, the usability of telehealth was evaluated through the TUQ, which was developed in 2016 to evaluate the usability of telehealth implementation and services. The TUQ consists of 21 items and 6 components: Usefulness, Ease of Use and Learnability, Interface Quality, Interaction Quality, Reliability, and Satisfaction. The patients were asked to respond to each item on a 5-step Likert scale, and the ratio of the sum of scores for each component to its total possible score was calculated.

### Statistical analysis

For statistical analysis, SPSS 29 was employed (IBM Corp, Armonk, NY, USA). Because the Shapiro–Wilk test revealed a lack of conformity to a normal distribution, a Wilcoxon signed-rank test was employed to compare the results between baseline and 6 months. Additionally, the Mann–Whitney *U* test and Pearson’s χ^2^ test were used to analyse the results of the PDQ-39. Values of *p* < 0.05 were considered to indicate statistical significance. The relationship between the amount of change in the PDQ-39 and TUQ was analysed by Spearman’s rank correlation coefficient. Also, the effect between whether or not the PDQ, ADL, or IADL has improved and TUQ was performed by Wilcoxon signed-rank test.

## RESULTS

Out of 63 patients who gave consent to participate in this study, 50 patients attended telerehabilitation every time for 6 months. For 6 patients who participated less frequently, the reasons were as follows: 2 patients reported that conflicts with their welfare schedules made participation difficult, 1 patient frequently forgot to participate unintentionally, 1 patient had delays in participation due to the time required from consent to the start of participation, 1 patient was unable to continue participating due to computer malfunctions during the study period, and 1 patient faced complication with online access. Four patients never participated in the telerehabilitation, and 3 were hospitalized for reasons unrelated to PD during the study period, which made it challenging for them to complete the questionnaire after 6 months. As a result, these 7 patients were excluded, leaving 56 patients for the analysis. The demographic and clinical characteristics of the 56 patients (mean age 73.8 ± 5.5 years, disease duration 6.0 ± 6.8 years, and HY stage 2.3 ± 1.0) are given in Table I. Throughout the study period, patients had no restrictions on medication changes; however, the majority (78.6%) maintained their drug dosages. At study onset, the most common troublesome symptoms among patients were postural abnormality, bradykinesia, and tremor (Table II). Among non-motor symptoms, constipation and frequent urination were frequent symptoms that troubled patients. Thirty-seven patients (66.1%) were aware of a decrease in physical activity during the COVID-19 pandemic and, among these, 91.9% responded that telerehabilitation helped them to develop an exercise routine after 6 months of participation (Table III). 91.1% of patients expressed satisfaction with the telerehabilitation programme; 50.0% of patients reported beneficial effects on their limb movement, while an even larger proportion, 71.4%, recognized the positive impact on their emotional well-being. Additionally, 73.2% reported that engaging in telerehabilitation together with other patients suffering from the same illness was a positive experience. Only 19.6% of participants felt that telerehabilitation helped to prevent an increase in medication, and just 19.6% reported that telerehabilitation had a positive impact on patients’ participation in social activities. Approximately half of the patients experienced improvements in ADL and IADL after 6 months (Table SIII). Among the subdomains, the most prominent improvement was observed in the domain of gait mobility.

**Table I:** Clinical characteristics of Parkinson’s disease (PD) patients at baseline

Characteristics	Values
Gender (male/female)	31/25
Age (years old)	73.8 ± 5.5 (57–90)
Body mass index (kg/m^2^)	21.9 ± 3.0 (16.2–29.6)
Hoehn and Yahr stage	2.3 ± 1.0 (1–4)
Disease duration (years)	6.0 ± 6.8 (0–42)
No change of anti-PD dosage during 6 months (%)	78.6%

**Table II:** Ranking of the 10 most bothersome Parkinson’s disease-related symptoms at baseline

Rank	Symptoms	Total score	First choice (%)	Second choice (%)	Third choice (%)
1	Postural abnormality	47	19.6	7.1	10.7
2	Bradykinesia	46	17.9	7.1	14.3
3	Tremor	34	17.9	1.8	3.6
4	Constipation	30	10.7	7.1	7.1
5	Postural instability	26	3.6	14.3	7.1
6	Gait disturbance	20	1.8	12.5	5.4
7	Frequent urination	18	1.8	10.7	5.4
8	Freezing of gait	18	7.1	5.4	0.0
9	Fatigue	17	7.1	1.8	5.4
10	Rigidity	15	1.8	7.1	7.1

**Table III:** Results of a self-report questionnaire on the effectiveness of telerehabilitation

Question	Yes (%)	No (%)	Neither (%)
The coronavirus disease 2019 pandemic has decreased my physical activity	66.1	26.8	7.1
Telerehabilitation helped me to develop an exercise routine (*n* = 37)	91.9	2.7	5.4
I am satisfied with the telerehabilitation	91.1	0	8.9
Telerehabilitation has a positive influence on my limb movement	50.0	1.8	48.2
Telerehabilitation has positive effects on my emotions	71.4	0.0	28.6
Telerehabilitation helps me avoid increases in medication dosage	19.6	25.0	55.4
Telerehabilitation had a positive impact on patients’ participation in social activities	19.6	3.6	76.8
It is good to having engaged in telerehabilitation together with patients suffering from the same illness	73.2	1.8	25.0

In a comparison between the baseline and 6-month administrations of the PDQ-39, a significant increase was evident in the total score. No statistically significant differences were detected in the subdomains, although the scores tended to be higher after 6 months (Table IV). Although the PDQ-39 score showed significant worsening in the total group of 56 patients, 19 out of 56 patients showed improvement of 1 or more points on the PDQ-39. In order to explore the possible effectiveness of telerehabilitation, we chose to take a closer look at the results of these 19 patients. For these patients, improvements were prominent in the subdomains of Mobility, Activities of Daily Living, Stigmatization, Social Support, and Body Discomfort (Table V).

**Table IV:** Changes in Parkinson’s Disease Questionnaire-39 (PDQ-39) scores between baseline and 6 months (*n* = 56)

PDQ-39	Baseline	After 6 months	*p*-value
Total	35.1 ± 19.7	38.7 ± 21.1	0.039*
Mobility	12.8 ± 10.1	13.9 ± 9.5	0.19
Activities of Daily Living	5.3 ± 4.7	6.0 ± 4.6	0.15
Emotional Well-being	5.6 ± 4.4	5.7 ± 4.3	0.71
Stigmatization	2.7 ± 2.1	2.8 ± 2.4	0.60
Social Support	1.1 ± 1.4	1.1 ± 1.6	0.89
Cognition	4.0 ± 2.8	4.8 ± 3.3	0.036*
Communication	1.2 ± 1.8	1.6 ± 2.3	0.13
Body Discomfort	2.4 ± 2.4	2.8 ± 2.7	0.19

* *p* < 0.05 by Wilcoxon signed-rank test.

**Table V:** Changes in Parkinson’s Disease Questionnaire-39 (PDQ-39) scores between baseline and 6 months in the group showing improvement of 1 or more points on the Parkinson’s Disease Questionnaire-39 (*n* = 19)

PDQ-39	Baseline	After 6 months	*p*-value
Total	44.8 ± 21.0	34.6 ± 17.8	< 0.001*
Mobility	17.4 ± 10.9	14.5 ± 9.1	0.008*
Activities of Daily Living	7.2 ± 5.2	5.2 ± 4.2	0.021*
Emotional Well-being	6.7 ± 5.4	4.9 ± 3.7	0.097
Stigmatization	3.4 ± 2.4	2.2 ± 2.0	0.032*
Social Support	1.5 ± 1.7	0.7 ± 1.0	0.028*
Cognition	4.2 ± 3.1	3.6 ± 2.2	0.23
Communication	1.2 ± 1.8	1.2 ± 1.7	0.86
Body Discomfort	3.2 ± 2.3	2.3 ± 2.6	0.027*

* *p* < 0.05 by Wilcoxon signed-rank test.

Regarding the TUQ, higher scores were obtained for Usefulness (76.1%), Ease of Use and Learnability (73.5%), Interface Quality (75.4%), and Satisfaction and Future Use (82.2%) (Table VI). However, scores for Interaction Quality and Reliability were slightly lower (67.6% and 66.1%, respectively).

**Table VI:** Satisfaction with telerehabilitation as evaluated by the Telehealth Usability Questionnaire

Components	Ratio of each item to total score (%)
Usefulness	76.1
Ease of Use and Learnability	73.5
Interface Quality	75.4
Interaction Quality	67.6
Reliability	66.1
Satisfaction and Future Use	82.2

Although the relationship between changes in PDQ-39 and each component of the TUQ was investigated, no significant correlations were found. Additionally, when PDQ-39 scores were divided into 2 groups – those with improvement and those with worsening – and compared with TUQ scores, no significant differences were observed. However, when TUQ scores were compared based on improvement in ADL, the group reporting improvement in ADL rated all TUQ components as more favourable than the group without improvement in ADL, with significant differences noted in Usefulness, Interface Quality, Reliability, Satisfaction, and Future Use (Table VII). Similarly, in the comparison of TUQ scores with and without improvement in IADL, all components were rated more favourably in the group with improvement of IADL, but only Usefulness showed a significant difference (Table VIII).

**Table VII:** Relationship between improvement in activities of daily living (ADL) and usability

Components of TUQ	Improvement in ADL		
	Yes (%) (*n* = 32)	No (%) (*n* = 24)	*p*-value
Usefulness	81.7 ± 12.4	68.6 ± 15.0	0.002
Ease of Use & Learnability	76.9 ± 16.8	68.9 ± 22.3	0.2
Interface Quality	80.0 ± 12.2	69.4 ± 15.9	0.01
Interaction Quality	70.3 ± 14.9	64.0 ± 13.5	0.2
Reliability	71.0 ± 15.7	59.4 ± 14.6	0.006
Satisfaction and Future Use	85.5 ± 12.1	77.9 ± 12.7	0.03

TUQ: Telehealth Usability Questionnaire.

**Table VIII:** Relationship between improvement in instrumental activities of daily living (IADL) and usability

Components of TUQ	Improvement in IADL		
	Yes (%) (*n* = 22)	No (%) (*n* = 34)	*p*-value
Usefulness	83.0 ± 11.8	71.6 ± 15.2	0.007
Ease of Use & Learnability	77.0 ± 18.0	71.2 ± 20.4	0.3
Interface Quality	79.5 ± 13.7	72.8 ± 15.0	0.1
Interaction Quality	69.1 ± 13.8	66.6 ± 15.2	0.6
Reliability	68.5 ± 15.0	64.5 ± 16.9	0.4
Satisfaction and Future Use	84.8 ± 12.5	80.6 ± 12.9	0.3

TUQ: Telehealth Usability Questionnaire.

## DISCUSSION

Telerehabilitation may have the potential to improve emotional well-being through physical activity. Additionally, many participants have reported positive experiences when participating in telerehabilitation together with other patients with the same disease, which could potentially have contributed to a reduction in feelings of loneliness and an improvement in emotional well-being in this study. Social isolation can lead to a negative spiral by triggering a decrease in physical activity and an exacerbation of stress, which in turn can contribute to the worsening of both motor and non-motor symptoms. In recent years, the COVID-19 epidemic has exacerbated this negative spiral. (14). In this study as well, 70% of the patients were aware that their physical activity had decreased during the COVID-19 pandemic. The majority of them reported that telerehabilitation had contributed to their acquisition of exercise habits, and we presume that this helped them to escape the negative spiral associated with social isolation. After events such as the COVID-19 pandemic come to an end, face-to-face rehabilitation is preferable in many ways (e.g., real in-person social interactions, the presence of therapists). On the other hand, we believe that telerehabilitation can be beneficial for patients with severe symptoms who have difficulty going to medical facilities or for those living far from such facilities. We have also found that many patients feel mental benefits from being in the company of others with the same illness, and we believe that telerehabilitation may be beneficial for their mental health. We believe that telerehabilitation can be one of the methods for providing rehabilitation, alongside in-person rehabilitation and in-home rehabilitation.

In our study, the satisfaction rate with telerehabilitation was very high at 91.1%. Across all 56 patients, the PDQ-39 score significantly worsened over 6 months (*p* = 0.04) (see Table IV). Despite these results, it is interesting that patients’ satisfaction with telerehabilitation remained high. According to the responses to the self-administered questionnaire we created (see Table III), a high percentage of respondents reported positive effects on their emotions and favourable feelings for participating in telerehabilitation together with others suffering from the same illness. Therefore, we speculate that positive effects on emotional well-being and the reduction of loneliness contributed to high satisfaction with the telerehabilitation programme. Additionally, various attempts were made to ensure that PD patients continued to participate without experiencing monotony. First, we changed the rehabilitation content appropriately every 2 weeks. Second, the attending physician always participated. Third, all content was provided free of charge to patients. Fourth, the physiotherapists and attending physicians delivered mini-lectures encompassing a diverse array of topics, ranging from practical strategies for daily life and basic knowledge concerning PD to the most recent advancements in treatments. We believe that these attempts and the opportunity to connect with others facing the same disease played a pivotal role in fostering a notable level of patient satisfaction.

Regarding ADL/IADL, approximately half of the patients perceived improvement, particularly in gait mobility, despite the fact that the exercises were performed while seated. Even through telerehabilitation, it was suggested that automatic range of motion training and trunk muscle strength training may potentially contribute to the improvement of ADL and gait function in some patients. The baseline survey revealed that the most troublesome symptom among participating PD patients was postural abnormalities. Therefore, it is believed that in order to improve ADL and IADL, it is necessary to conduct more rehabilitation programmes with a focus on addressing postural abnormalities.

Although some previous reports have demonstrated improvement in QOL (8, 15), unfortunately, a significant worsening of the PDQ-39 total score was observed between baseline and 6 months later in this study (see Table IV). Nineteen out of 56 patients showed an improvement of at least 1 point on PDQ-39, while 37 patients experienced no change or worsening. One possible reason for this discrepancy is that the inclusion criteria in this study were not rigorous, with inclusion of PD patients across all disease stages and with varying degrees of severity ranging from mild to severe. In contrast, previous studies were conducted on younger patients with an average age of 60 years for shorter duration of rehabilitation and with a more intensive approach compared with our study (8, 15). In addition, some patients experienced a notable decline in their QOL due to worsening comorbidities, such as lumbar spinal canal stenosis and severe autonomic dysfunction. Although a small number of people, 19 patients who reported improvement in their QOL exhibited a trend towards enhancements across many subdomains in the PDQ-39 (see Table V). This trend aligns with findings from previous reports (15), which suggests that telerehabilitation could potentially contribute to an improved quality of life, while factoring out the effects of comorbidities and ageing.

In our study, the specialized questionnaire called the TUQ was employed for evaluating the usability of telehealth implementation and services. PD patients expressed substantial satisfaction in terms of Usefulness and Ease of Use and Learnability, because access was easy and no hospital visit was required. In our present study, upon initiating internet-based rehabilitation for elderly patients, there was a technical problem with the initial settings. To overcome this issue, many participants received help from their spouses and children, while others received support from the rehabilitation staff. Interaction Quality and Reliability were limited, because only 1 physical therapist provided instruction to a group of PD patients, although the physical therapist provided guidance to patients as much as possible.

### Limitations

Several remaining limitations to our study should be noted. First, patient-reported outcomes were obtained only from patients participating in telerehabilitation. Second, an intention-to-treat analysis would be most desirable; however, 7 out of 63 patients were excluded from the analysis. Three patients were hospitalized for reasons unrelated to PD and 4 patients never participated in the telerehabilitation. Third, telerehabilitation was offered only to patients with available hardware and software, and this may have affected satisfaction. Fourth, there were no comparative data for in-person rehabilitation. Fifth, we were unable to observe changes in motor symptoms as measured by the MDS-Unified Parkinson’s Disease Rating Scale Part III, or to investigate details on mood. Sixth, the reason for this is that 7 of the 63 students (the 3 who required hospitalization and the 4 who never participated during the study) dropped out during the course of the study. Seventh, as only 56 patients were enrolled, and the follow-up period was only 6 months, the impact of extensive and long-term telerehabilitation needs to be investigated in detail in the future.

### Conclusion

Satisfaction with telerehabilitation was notably high in our patients with PD, especially in terms of its positive effects on emotional well-being. The usability of telerehabilitation was also found to be high. We cannot have conclusive remarks about the effectiveness.

## Supplementary Material




