# Supplementary material for: SATISFACTION, EFFECTIVENESS, AND USABILITY OF TELEREHABILITATION FOR PARKINSON’S DISEASE PATIENTS
Source: J Rehabil Med. 2025 Jan 3;57:39819. doi: 10.2340/jrm.v57.39819 (PMC11681136; doi:10.2340/jrm.v57.39819)
Supplement: Supplementary file 1 [file JRM-57-39819-s1.pdf]

Supplementary material has been published as submitted. It has not been copyedited, or typeset by Journal of Rehabilitation Medicine

Table SI. The content of the telerehabilitation for Parkinson's disease patients

	**Type**
**Basic exercises**	
Stretching	Sustained stretching or dynamic stretching before exercise.
Strengthening	Resistance training with body weight for major muscle groups of the upper and lower extremities and trunk.
Aerobic	Continuous, rhythmic exercises such as stepping and arm swinging in a seated position.
**Multimodal exercise**	
Rhythmic exercises	Rhythmic movement of the extremities to music in seated position, including diadochokinesis.
ADL related exercises	Exercises that incorporate movements similar to basic activities of daily living such as dressing, toilet use, and standing and sitting.
Dual-task exercise	Motor and cognitive dual-task exercises in which the extremities are moved rhythmically while performing calculation and stroop tasks.
**Swallowing exercises**	Exercises to increase range of motion and strength of the lips, tongue, and neck muscles.
**Speech exercises**	Practice speaking and singing loudly.

* All programs consist of seated exercises and do not require any special equipment.

ADL: activities of daily living

Table SII. The TIDieR (Template for Intervention Description and Replication) Checklist

Information to include when describing an intervention and the location of the information

Item number	Item	Where located ** Primary paper (page or appendix number)	Other † (details)
	**BRIEF NAME**		
**1.**	Provide the name or a phrase that describes the intervention.	1	
	**WHY**		
**2.**	Describe any rationale, theory, or goal of the elements essential to the intervention.	5	
	**WHAT**		
**3.**	Materials: Describe any physical or informational materials used in the intervention, including those provided to participants or used in intervention delivery or in training of intervention providers. Provide information on where the materials can be accessed (e.g. online appendix, URL).	5, 6, Table SII	
**4.**	Procedures: Describe each of the procedures, activities, and/or processes used in the intervention, including any enabling or support activities.	6	
	**WHO PROVIDED**		
**5.**	For each category of intervention provider (e.g. psychologist, nursing assistant), describe their expertise,	6, 10	

	background and any specific training given.		
	**HOW**		
**6.**	Describe the modes of delivery (e.g. face-to-face or by some other mechanism, such as internet or telephone) of the intervention and whether it was provided individually or in a group.	_6, Figure SI_	____________
	**WHERE**		
**7.**	Describe the type(s) of location(s) where the intervention occurred, including any necessary infrastructure or relevant features.	________6____	____________
	**WHEN and HOW MUCH**		
**8.**	Describe the number of times the intervention was delivered and over what period of time including the number of sessions, their schedule, and their duration, intensity or dose.	________6____	____________
	**TAILORING**		
**9.**	If the intervention was planned to be personalised, titrated or adapted, then describe what, why, when, and how.	________6____	____________
	**MODIFICATIONS**		
**10.**[‡]	If the intervention was modified during the course of the study, describe the changes (what, why, when, and how).	_______6, 10____	____________
	**HOW WELL**		
**11.**	Planned: If intervention adherence or fidelity was assessed, describe how and by whom, and if any strategies were used to maintain or improve fidelity, describe them.	______6, 7, 8, 10	____________

**12.**[‡]	Actual: If intervention adherence or fidelity was assessed, describe the extent to which the intervention was delivered as planned.	7, 8	

** **Authors -** use N/A if an item is not applicable for the intervention being described. **Reviewers** – use '?' if information about the element is not reported/not sufficiently reported.

† If the information is not provided in the primary paper, give details of where this information is available. This may include locations such as a published protocol or other published papers (provide citation details) or a website (provide the URL).

ǂ If completing the TIDieR checklist for a protocol, these items are not relevant to the protocol and cannot be described until the study is complete.

* We strongly recommend using this checklist in conjunction with the TIDieR guide (see *BMJ* 2014;348:g1687) which contains an explanation and elaboration for each item.

* The focus of TIDieR is on reporting details of the intervention elements (and where relevant, comparison elements) of a study. Other elements and methodological features of studies are covered by other reporting statements and checklists and have not been duplicated as part of the TIDieR checklist. When a **randomised trial** is being reported, the TIDieR checklist should be used in conjunction with the CONSORT statement (see www.consort-statement.org) as an extension of **Item 5 of the CONSORT 2010 Statement.** When a **clinical trial protocol** is being reported, the TIDieR checklist should be used in conjunction with the SPIRIT statement as an extension of **Item 11 of the SPIRIT 2013 Statement** (see www.spirit-statement.org). For alternate study designs, TIDieR can be used in conjunction with the appropriate checklist for that study design (see www.equator-network.org).

Table SIII. The percentage of improvement in activities of daily living/ instrumental activities of daily living after 6 months

	N (%)		N (%)
Improvement of ADL	32 (57.1)	Improvement of IADL	22 (39.3)
Improvement of ADL subdomain	N (%)	Improvement of IADL subdomain	N (%)
Mobility	25 (44.6)	Housekeeping	7 (12.5)
Dressing	11 (19.6)	Transportation	6 (10.7)
Stairs	7 (12.5)	Shopping	6 (10.7)
Feeding	5 (8.9)	Food preparation	4 (7.1)
Grooming	2 (3.6)	Managing medications	2 (3.6)
Transfers	2 (3.6)	Communication with others	1 (1.8)
Toilet use	1 (1.8)	Laundry	1 (1.8)
Bathing	0	Managing finances	0
Bowels	0		
Bladder	0		

ADL: Activities of Daily Living; IADL: Instrumental Activities of Daily Living.
