# Supplementary material for: SATISFACTION, EFFECTIVENESS, AND USABILITY OF TELEREHABILITATION FOR PARKINSON’S DISEASE PATIENTS
Source: J Rehabil Med. 2025 Jan 3;57:39819. doi: 10.2340/jrm.v57.39819 (PMC11681136; doi:10.2340/jrm.v57.39819)

Supplementary material has been published as submitted. It has not been copyedited, or typeset by Journal of Rehabilitation Medicine

Fig. S1. A telerehabilitation using the Zoom Meeting platform. One physical therapist provides rehabilitation instruction to several patients with Parkinson's disease

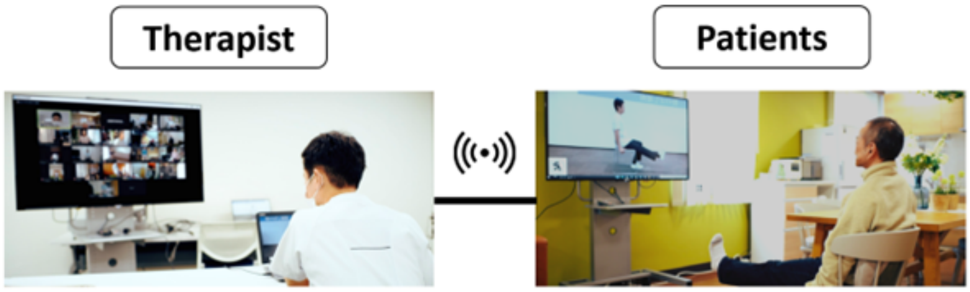

Supplement: Supplementary file 2 [file JRM-57-39819-s2.pdf]
